# TLR ligands, but not modulators of histone modifiers, can induce the complex immune response pattern of endotoxin tolerance in mammary epithelial cells

**DOI:** 10.1177/1753425916681076

**Published:** 2016-12-05

**Authors:** Juliane Günther, Wolfram Petzl, Holm Zerbe, Hans-Joachim Schuberth, Hans-Martin Seyfert

**Affiliations:** 1 Leibniz Institute for Farm Animal Biology, Institute for Genome Biology, Dummerstorf, Germany; 2 Clinic for Ruminants with Ambulance and Herd Health Services, Centre for Clinical Veterinary Medicine, LMU Munich, Munich, Germany; 3 Immunology Unit, University of Veterinary Medicine Foundation, Hannover, Germany

**Keywords:** Endotoxin tolerance, epigenetic mechanisms, mammary epithelial cells, mastitis, immune modulation

## Abstract

Excessive stimulation of the TLR4 axis through LPS reduces the expression of some cytokine genes in immune cells, while stimulating the expression of immune defense genes during a subsequent bacterial infection. This endotoxin tolerance (ET) is mediated via epigenetic mechanisms. Priming the udder of cows with LPS was shown to induce ET in mammary epithelial cells (MEC), thereby protecting the udder against reinfection for some time. Seeking alternatives to LPS priming we tried to elicit ET by priming MEC with either lipopeptide (Pam2CSK4) via the TLR2/6 axis or inhibitors of histone-modifying enzymes. Pre-incubation of MEC with Pam2CSK4 enhanced baseline and induced expression of bactericidal (β-defensin; *SLPI*) and membrane protecting factors (*SAA3*, *TGM3*), while reducing the expression of cytokine- and chemokine-encoding genes (*TNF*, *IL1β*) after a subsequent pathogen challenge, the latter, however, not as efficiently as after LPS priming. Pre-treating MEC with various inhibitors of histone H3 modifiers (for demethylation, acetylation or deacetylation) all failed to induce any of the protective factors and only resulted in some dampening of cytokine gene expression after the re-challenge. Hence, triggering immune functions via the TLR axis, but not through those histone modifiers, induced the beneficial phenomenon of ET in MEC.

## Introduction

Inflammation and infection of the udder (mastitis) is a frequent and highly relevant disease in dairy farming.^1^ Infections of Gram-negative pathogens, such as *Escherichia coli*, frequently cause severe inflammation and clinical symptoms.^2,3^ The risk of suffering from a new udder infection is by far highest during the first 2 wk after calving,^4^ hence only during a limited period of time. It is therefore appealing to search for treatments that protect the udder against infection during that critical and timely limited period. Such treatments are best not based on the application of antibiotics. We have previously demonstrated the principle feasibility of such an approach by showing that a short-term (12 h) local application of a low dose of LPS (1 µg/udder quarter) into the udder of mid-lactating cows reliably protected against new infection with *E. coli* for several days. The treatment provided a longer lasting (10 d) protection against severe systemic symptoms in the case of a successful reinfection.^5^

We found that LPS priming induced ‘endotoxin tolerance’ (ET) in mammary epithelial cells (MEC) was the likely cause underpinning the reduced infection probability and milder symptoms during a subsequent reinfection.^6^ MEC are the most abundant cells in the lactating udder,^7^ and their pathogen species-specific immune reaction norm determines the immune response of the udder early after infection.^8^

The phenomenon of ET characterizes reduced immune responsiveness of immune cells to a LPS challenge subsequent to a previous exposure to *E. coli* or LPS.^9^ ET is induced during sepsis, for example through excessive LPS-mediated TLR4 stimulation.^10^ ET has dual key features. On the one hand, it reduces the risk of immune pathology during the subsequent LPS challenge by dampening—or even abrogating—the induction of pro-inflammatory cytokine- and chemokine-encoding genes. On the other hand, it reduces the probability of renewed colonization by invading pathogens through sustaining increased expression of bactericidal factors.^6,11^ Key mechanisms underpinning ET physiology include chromatin remodeling and histone modifications at the promoters of relevant genes.^12^ Modifications of histone H3 through acetylation and the addition or removal of methyl groups are of pivotal importance.^13–16^ Such modifications, combined with DNA methylation, will regulate the access of key transcription factors to the promoters.^17^ Recruitment of members of the NF-κB factor family is crucial in this regard,^18^ as they are key regulators of immune functions.^19,20^ Recruitment of NF- κBp50 (NFKB1) is particularly relevant during ET,^21,22^ not least because this factor may recruit a repressome onto the target promoters subsequent to excessive TLR4 signaling.^23^

Excessive stimulation of other TLR receptors, such as TLR2 or TLR5, may induce ‘cross-tolerance’. This is an immune refractory state quite similar to ET and may not only be in elicited in professional immune cells,^24–26^ but also in alveolar epithelial cells.^27^

Based on our well-established model of primary bovine MEC (pbMEC),^8,28^ we wanted to identify alternatives for LPS to protect the udder successfully against reinfection, as LPS is the prototypical ‘endotoxin’ and as such hardly an acceptable pharmaceutical. On the one hand, we explored the value of the synthetic TLR2/6 ligand, Pam2CSK4,^29^ as a model substance for derivatives of bacterial lipopeptides or lipoproteins to induce ET in MEC. On the other hand, we examined if pharmaceutically approved inhibitors of different histone-modifying enzymes might also be capable of inducing ET in these cells. If successful, then using such already medically approved drugs might offer realistic opportunities to develop applicable novel interventions against mastitis.

Marking histone H3 through the differential addition of methyl or acetyl groups is accomplished by different classes of enzymes. Histone acetyltransferases (HATs) may acetylate H3 to enhance gene expression.^13^ Hence, we assumed that blocking histone deacetylases (HDACs) might result in increased baseline expression of late secondary response genes encoding bactericidal and membrane protective factors, while blocking the HATs should reduce the extent of gene induction after the re-challenge. This could be particularly desirable for confining the response of the immediate early pro-inflammatory cytokine- and chemokine-encoding genes. We therefore tested suberoylanilide hydroxamic acid (also known as SAHA or Vorinostat)^13^ and trichostatin A (TSA)^30^ as small-molecule inhibitors of the HDACs. S2101 and GSK-J4 served to inhibit lysine-specific histone demethylases 1A or 6B (JMJD3),^13,31^ and C646 to block the HAT CREB-binding protein (p300/CBP).^32^ Parameters for ET induction were the priming and re-challenge-related modulation of the mRNA concentration of a selection of master cytokines (TNF-α, IL-1α, IL-β, IL-6)^33–35^ and chemokines (CCL2, CCL5, CCL20, CXCL8, CXCL2);^36^ and of factors protecting membranes [serum amyloid A3 (SAA3)^37^ and transglutaminase 3 (TGM3)^38^] and fighting off bacteria (β-defensin LAP,^39^ NO synthase NOS2A,^40^ secreted leukocyte protease inhibitor SLPI,^41^ S100A9^42^). We also surveyed the expression of key transcription factors and transcription regulators (NF-κBp50; NF- κBIζ as an LPS-inducible regulator,^43^ acting sometimes as an antagonist to NF- κBp50/p65 function;^44^ nuclear receptor subfamily 4 group A member 2, NR4A2—more widely known as Nurr1—dampening overshooting inflammation^45^); of auxiliary factors and regulators of TLR signaling (CD36,^46^ CD40,^47^ SIGIRR^48^); and of the histone lysine-specific demethylase 6B (KDM6B), also known as JMJD3.^49^

We found that priming with Pam2CSK4 can, indeed, induce ET in MEC, almost as efficiently as LPS but that any of the small-molecular inhibitors of histone modifiers would only dampen immune gene expression, but not significantly enhance expression of any of the protective factors.

## Material and methods

### Cell culture procedure, priming and challenge with heat-inactivated E. coli particles

pbMEC were prepared as described.^50^ Tissues were obtained from udders of healthy first lactating cows slaughtered at our local abattoir, complying with all pertinent ethical and legal requirements (EU license ES1635). Cultivation of pbMEC in RPMI 1640 (Biochrom, Berlin, Germany) supplemented with insulin, prolactin, dexamethasone and 10% FCS (PAN-Biotech, Aidenbach, Germany) was as described in detail previously.^28^

**Figure 1.**
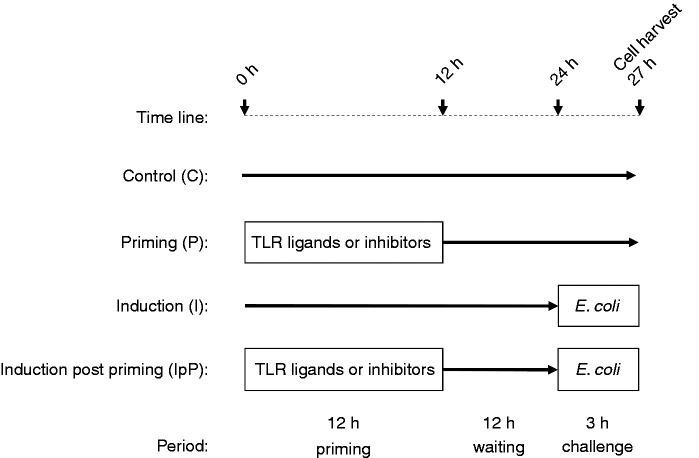
Schematic diagram of the experimental setting.

Priming and challenge experiments were designed following our previously published LPS priming procedure (see scheme in Figure 1).^6^ Briefly, for priming experiments (P) the cells were incubated for 12 h (‘primed’) either with TLR ligands [highly purified LPS prepared from *E. coli* strain 1303,^6^ or Pam2CSK4 (InvivoGen, Toulouse, France)] or inhibitors of histone-modifying enzymes [S2101 (Calbiochem, Merck Millipore, Darmstadt, Germany), C646 (Sigma-Aldrich, Munich, Germany) or SAHA (Sigma-Aldrich)]. It was validated that the LPS preparation used in this study did not activate TLR2 (Supplementary Figure S1). Control cells were cultivated for 12 h in normal growth medium (GM). After 12 h, all cells were washed three times with PBS and subsequently cultivated for an additional 15 h. To evaluate the effect of priming upon a re-stimulation [induction post-priming experiments (IpP)] cells were first primed for 12 h with the respective substance or cultivated in plain GM as controls, washed three times with PBS and cultivated for 12 h in GM without any priming substance added (waiting period). Subsequently, we supplemented each of these cultures (primed or un-primed controls) for 3 h with 30 µg/ml heat-killed *E. coli* 1303 particles. All cells were harvested at 27 h and total RNA was prepared. The heat-killing procedure for the *E. coli* pathogens was just as previously described.^51^

### Vitality assay

Potential cytotoxicity of inhibitors targeting histone-modifying enzymes (S2101, C646 and SAHA) on MEC was tested with a MTT assay (Cell Proliferation Kit I; Roche, Penzberg, Germany). Briefly, cells were incubated for 12 h in normal growth medium or in medium containing three different concentration of the substances (S2101: 5 µM, 50 µM or 500 µM; C646: 2 µM, 20 µM or 200 µM; SAHA: 20 nM, 100 nM or 1000 nM). The number of viable cells were analyzed with the colorimetric MTT assay as recommended by the manufacturer. Data are represented relative to the value from untreated control cultures set as 100% (Supplementary Figure S2).

### RNA extraction and mRNA quantification

Total RNA was extracted with the TRIZOL reagent (Invitrogen, Thermo Fisher Scientific, Waltham, MA, USA). Synthesis of cDNA (Superscript II; Invitrogen) from an input of 75 ng total RNA/sample and quantification of mRNA by real-time PCR with the Fast-Start SYBER Green I kit and the LightCycler II instrument (Roche, Penzberg, Germany) was performed as described.^52^ Relative copy numbers were determined by titration against external standards consisting of dilution series of plasmids (10^6^–10 copies) harboring the respective amplicon. Normalization was performed against the copy numbers of the reference gene chloride intracellular channel 1 (*CLIC1*). The expression of this gene is not regulated in pbMEC in such experimental settings.^53^ Sequences of primer pairs are listed in Supplementary Table S1.

To calculate the priming caused modulation of the mRNA levels, we set as 1.0 the *E. coli* stimulation-caused fold induction as recorded from the un-primed control cells and expressed the same values as recorded from the primed cultures as decimals hereof. The inverse of these values was presented in case they were smaller than 1.0 (as encountered in the experimental setting of IpP). If, for example, *E. coli* induced the expression by 10-fold in the unprimed controls and only by twofold in the primed cells, then this would be equivalent to only 0.2-fold stimulation in the IpP situation. To better visualize this difference, we presented the negative value of the inverse, e.g. 1/0.2 = –5-fold.

### Statistical analysis

Data were analyzed using GraphPad Prism Version 5 (GraphPad Software, Inc., La Jolla, CA, USA). The Wilcoxon test was applied to evaluate the effect of Pam2CSK4 priming on the basal (P) and *E. coli* induced (IpP) expression of multiple candidate genes. Repeated-measures ANOVA and Dunnett’s multiple comparison tests were conducted to estimate the priming effect of inhibitors targeting histone-modifying enzymes (S2101, C646, SAHA) on basal (P) and *E. coli*-induced (IpP) gene expression.

## Results

We evaluated in a first round of experiments if the TLR2/TLR6 heterodimer ligand Pam2CSK4 would elicit ET in the pbMEC. We used, in principle, our previous experimental setting with which we had proven the efficacy of LPS priming in eliciting ET in MEC (Figure 1).^6^ Cells were primed for 12 h with different concentrations of Pam2CSK4. Then the priming substance was washed away and the cells were either kept in fresh medium for another 15 h, or challenged 12 h after the wash for another 3 h with 30 µg/ml heat-killed *E. coli* 1303 particles. We know from many previous studies that this stimulus elicits a near-maximal immune response in MEC.^54^ LPS-challenged cultures were eventually run in parallel, serving as positive controls and allowing direct comparison of the efficacy of the different priming substances.

### Effect of priming via the TLR axis upon basal gene expression

**Figure 2.**
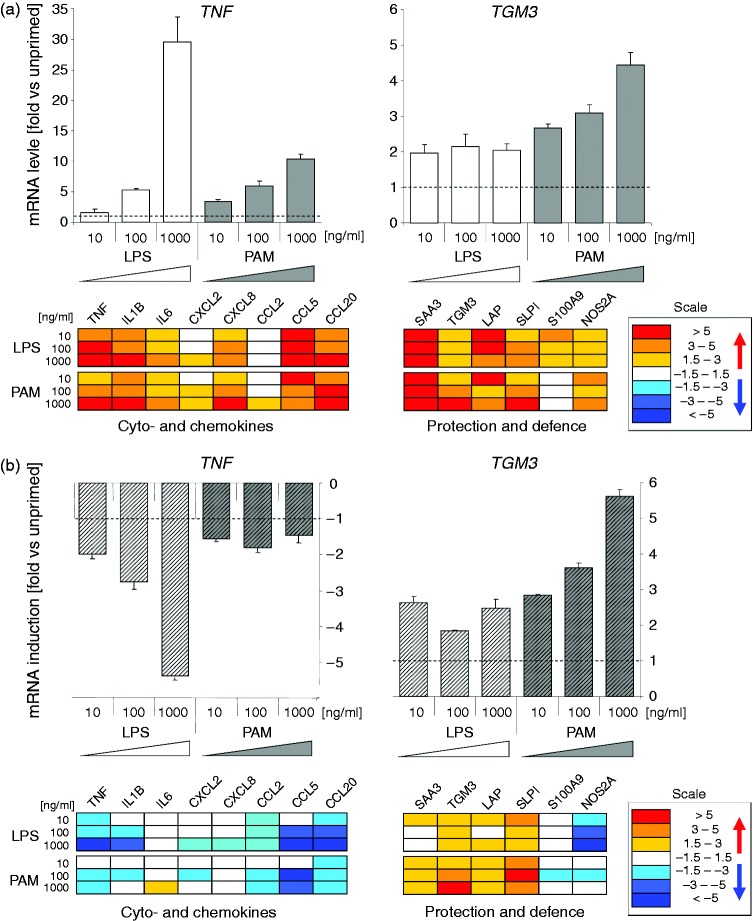
Dose dependence of LPS and Pam2CSK4 priming mediated modulated gene expression in pbMEC. (a) Upper diagrams: fold changes (ordinate) of the relative mRNA concentrations of TNF and TGM3 in response to priming alone for 12 h relative to un-primed control cultures (*cf.* Figure 1: ‘Control’). Below: heat map of the expression of other genes, recorded in the same experiment. The box shows the scale. Numerical values for these and all other analyzed genes are listed in Supplementary Table S2. (b) Same as above, but values are expressed relative to the extent of *E. coli* caused induction of the unprimed cultures set as 1.0. Values < 1 are presented as the negative of the inverse value. Data are mean values (± SEM) from two technical replica experiments of pbMEC derived from a single cow.

Analysis of the priming effect upon the steady-state basal level of gene expression is informative as almost all molecules require constant re-synthesis owing to their limited half lives. Priming related modulation of the expression of bactericidal factors is particularly relevant in this regard as their synthesis and secretion influences the chemical composition of the alveolar fluid, the niche in which an intruding pathogen would have to survive in. We found in the first screenings that Pam2CSK4 priming modulated in a dose-dependent fashion the baseline expression of most of our candidate pro-inflammatory cytokine and chemokine-encoding genes, quite similar in magnitude as LPS priming (Figure 2; Supplementary Table S2). These genes included *TNF*, *IL1β*, *IL6*, *CXCL2*, *CXCL8*, *CCL5* and *CCL20*. Moreover, it also enhanced the baseline expression of the group of late-responding secondary immune genes. This group of genes included those expressing not only the membrane-protecting factors *SAA3* and *TGM3*, but also the bactericidal factors β-defensin *LAP*, *SLPI*, *S100A9* and *NOS2A*. The effect of Pam2CSK4 on the expression of those genes with protective functions was, in tendency, even stronger than that of LPS. For example, Pam2CSK4 priming increased the mRNA concentration of NOS2A and SLPI by 5–6-fold as opposed to the 2.5–3-fold increase caused by LPS.

Neither LPS nor Pam2CSK4 priming substantially affected the basal mRNA expression of other groups of genes encoding potentially relevant transcription factors (NF-κBp50, NF-κBIζ, NR4A2), auxiliary factors and regulators of TLR signaling (CD36, CD40, SIGIRR), or the JMJD3 factor known to be key for crucial histone H3-modifications (Supplementary Table S2).

### Effect of priming via the TLR axis upon re-challenge-induced gene expression

Challenging previously primed cells after a 12 h waiting period in fresh medium with the heat-killed *E. coli* 1303 particles induced the expression of several of the pro-inflammatory genes to a lesser extent than recorded from the un-primed controls (Figure 2; Supplementary Table S2). However, we noted here a difference between the LPS- and Pam2CSK4-primed cells. LPS priming reduced the extent of re-stimulation-dependent induction of gene expression for most of these genes, in a dose-dependent fashion (*TNF, IL-1β, CXCL8, CCL20, CCL2, CCL5, CXCL2*), while Pam2CSK4 priming quenched the extent of their induction as well, but mostly to a lesser extent and independent of the concentration of the priming substance. For example, LPS priming with 1000 ng/ml quenched the re-challenge-induced mRNA concentration of TNF-α and IL-1β almost six- and fivefold (TNF-α and IL-1β, respectively), while priming the cells with Pam2CSK4 lowered these values to only 2–1.7-fold (Supplementary Table S2). This also applied to the expression NOS2A. This factor is not only a potent bactericide, but also an enhancer of inflammation.^40^ LPS priming dramatically reduced its re-challenged stimulated expression in a strongly dose-dependent fashion (<1/10th of the induction of the un-primed controls, at 1000 ng/ml LPS), whilst the dampening effect of Pam2CSK4 was much smaller and independent of the dose of the primer (–1.3 fold at 1000 ng/ml Pam2CSK4; Figure 2, Supplementary Table S2).

Pam2CSK4 priming, as well as LPS priming, enhanced upon re-challenge with *E. coli* 1303 particles the expression of the late-responding membrane protecting and bactericidal factors (Figure 2; Supplementary Table S2). Indeed, the effect of Pam2CSK4 priming exceeded in tendency that of LPS for this group of genes.

Neither of both priming substances modulated the re-challenge-induced expression of all other groups of genes encoding the candidate transcription factors, auxiliary factors or JMJD3.

### Validation that priming with Pam2CSK4 induces cross-tolerance in MEC

We repeated the priming experiment with the high dose (1000 ng/ml) of Pam2CSK4 using two more pbMEC preparations, each derived from a different cow. The priming effect was statistically significant for the basal expression of all the early and late immune response immune genes, supporting the previous observations (Table 1). Moreover, the re-challenge assays (IpP) confirmed that this priming regime dampens the pathogen-mediated expression of the cytokine- and chemokine-encoding genes together with that of NOS2A, while it enhances the expression of bactericidal and membrane-protective factors. Together, the data show that triggering the immune functions of the MEC via the TLR2/6 axis quite faithfully recapitulate the dual key aspects of LPS-triggered and TLR4 mediated endotoxin tolerance.

**Table 1.** Validation of the priming effect of the TLR2/6 ligand Pam2CSK4.

Gene	Priming^a^	*E. coli* induction post priming^b^
*TNF*	10.2 ± 3.0^c^	–1.3 ± 0.1
*IL1Β*	6.9 ± 1.8	–1.6 ± 0.1
*IL6*	25.2 ± 7.3	1.7 ± 0.4
*CXCL8*	6.0 ± 0.7	–1.2 ± 0.1
*NOS2A*	3.5 ± 0.5	–1.7 ± 0.2
*TGM3*	3.2 ± 0.7	3.9 ± 0.7
*SLPI*	10.8 ± 1.8	6.1 ± 1.4
*SAA3*	204.6 ± 106.7	3.7 ± 1.1
*LAP*	4.3 ± 0.2	2.7 ± 0.3

a Priming was with 1000 ng/ml of Pam2CSK4 for 12 h. ^b^*E. coli* induction post-priming was for 3 h (see Figure 1), both treatments were applied in the same experimental setting as explained in Figure 2. Gray underlay, significant change (*P* < 0.05, Wilcox signed rank test). ^c^Values are fold changes of mRNA concentrations relative to unprimed control cultures (means ± SEM from three different biological replica cultures, each assayed in duplicate).

### Screening for relevant small-molecule inhibitors of chromatin modifiers

We next examined the efficacy of five different inhibitors of histone-modifying factors for priming immune functions in MEC. Different concentrations (1–100 µM) of GSK-J4 inhibiting the H3K27 histone demethylase JMJD3 had been included into the previous set of experiments. GSK-J4 priming left the basal expression of most of the genes virtually unaltered and the extent of re-challenged-induced gene expression was barely modulated compared with the un-primed controls (data not shown). The HDAC inhibitors SAHA and TSA^30^ were found in pilot experiments to modulate quantitatively the expression of responsive genes to a similar extent. We therefore included into our further analysis SAHA as the only deacetylase inhibitor and used C646^32^ as an inhibitor of the histone acetyl-transferase and S2101 to inhibit the histone lysine demethylase.^13^ We validated that our pbMEC could tolerate the published physiologically relevant concentrations for SAHA (1–100 nM)^55^ for C646 (10–50 µM)^32,56^ and for S2101 (1–50 µM).^57^ It was reported that C646 would be inhibited by the addition of FCS to the medium,^56^ while others proved its physiological activity in µM concentrations in medium containing 10% FCS.^58^ We found that its application in serum-free medium stressed our pbMEC resulting in cell death, as indicated by poor cell growth and 10-fold reduced RNA yield per culture dish at high concentrations (50 µM). Moreover, omission of serum significantly alters the immune response of pbMEC.^53^ Hence, we decided to apply C646 in medium containing 10% FCS.

### Inhibitors of chromatin modifiers are dampeners rather than enhancers of immune gene expression in MEC

Based on the experiences gathered in those pilot experiments, we monitored the efficacy of priming immune functions in pbMEC in two biological replica experiments each assayed in duplicate using pbMEC cultures derived from different cows. None of these inhibitors significantly increased the basal expression of most of our candidate genes (Table 2). Blocking the demethylases through S2101 and HATs through C646 affected the basal mRNA concentration in tendency quite similarly resulting either in no change (IL-1β) or some down-regulation. Only the high concentration of C646 (20 µM) raised the level on the CXCL8 mRNA by 1.9-fold. Blocking the HDACs through SAHA resulted in even fewer changes of the basal mRNA concentrations. As exceptions, we found for SAHA that priming with the high concentration (100 nM) increased the level of SLPI mRNA by 1.8-fold. This SAHA concentration also increased the SAA3 mRNA concentration by fourfold. Yet, priming with Pam2CSK4 had consistently increased this level by far more than > 100 fold (Table 1). We found, as a rule, that the extent by which the three different classes of inhibitors modulated the basal mRNA levels was smaller by almost an order of magnitude than encountered after priming the cells with either of both TLR ligands.

**Table 2.**
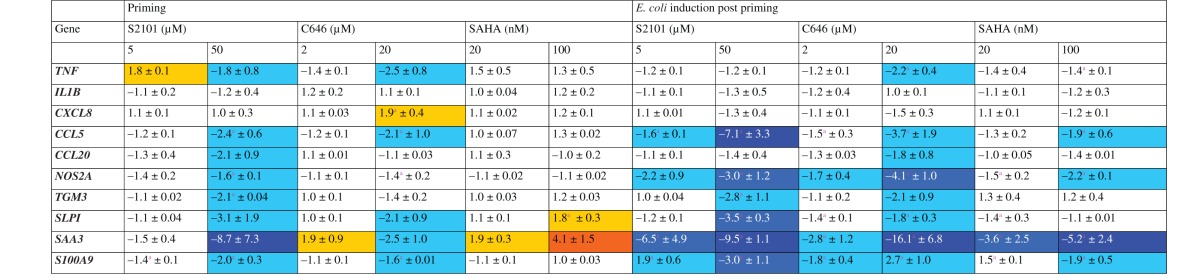
Heat map and numerical values of fold changes (mean ± SEM) of mRNA concentrations in pbMEC in response to 12 h pre-stimulation with modulators of histone modifiers followed by another 15 h resting in normal growth medium compared with un-stimulated cells or a 3 h *E. coli* challenge subsequent to a 12 h waiting period after priming (*E. coli* induction post priming).

Data are mean values ( ± SEM) from two biological replica experiments, each assayed in duplicate. They represent fold changes of the mRNA concentrations relative to unprimed control cultures. ^a^Priming significantly induced/repressed mRNA expression compared with un-stimulated (priming) or *E. coli*-challenged cells (*E. coli* induction post-priming)

Yellow:>+1.5; orange > + 3.0; cyan: <-1.5; mid-blue <; dark blue < -5.0.

Re-challenging the primed cells with the strong *E. coli* stimulus did not increase the expression of any of the genes but eventually caused only a down-regulation. Significantly, we found down- rather than up-regulation for the expression of almost all of the late secondary immune genes, including all those encoding protective and bactericidal factors (Table 2).

## Discussion

The goal of our ongoing work is to identify substances other than LPS that are capable of calibrating, after a local application, udder responsiveness to a repeated bacterial infection in a beneficial direction. The treatment should harness the bactericidal capacity of MEC and at the same time dampen their inflammation-eliciting potential. As a first step we tested here the efficacy of a variety of candidate substances for priming the immune responsiveness of pbMEC cultures and re-challenging them after a 12-h waiting period. We have previously validated this experimental setting by globally profiling the transcriptome of LPS-primed pbMEC,^6^ and also exemplified for IL-1Α,^51^ and the mRNA stability regulating factor tristetraprolin,^54^ that increased mRNA concentrations eventually correlate with increased protein concentrations in these model cells. In the present study, we recorded as a read-out the priming effect upon the re-challenge-caused induction of gene expression only at a single time point, at 3 h after the re-challenge. This is a compromise, considering the differential expression kinetics of primary and secondary immune-response genes. The mRNA concentrations of fast-responding primary immune genes (some cytokine- and chemokine-encoding genes)^59^ will peak within a few hours after a challenge,^54^ while those of some secondary response genes (e.g. LAP, SAA3) will steadily increase after the challenge, after an initial lag period of approximately 1 h.^54,60^ We know from many experiments that 3 h after the challenge the mRNA concentrations of the fast-reacting genes will still be elevated, while those of the slow-responding secondary genes only have started to rise.^53,54,60^ Therefore, the magnitude of the priming effect upon inducing the expression of the β-defensin *LAP*, for example, may have been considerably underestimated. Of note, we consider LAP expression only as an indicator and a paradigm for the more than 100 individual β-defensin-encoding genes of the bovine genome.^61^

Our first key observation is that priming immune functions in MEC via the TLR2/6 axis is possible and potentially as feasible as using LPS priming. Hence, induction of cross-tolerance through lipopeptides or other TLR2 ligands is possible in this cell type. This finding is novel for MEC and offers the possibility to eliciting ET in MEC with pure chemically synthesized molecules being much better defined than even the most highly purified LPS preparation derived from bacteria. It emerged, as second main result that inhibiting single enzymes involved in modifying histone H3 does not induce the complex features of endotoxin tolerance.

### Induction of cross-tolerance in MEC through TLR2 ligands may protect the udder from reinfection

Pam2CSK4 priming elicited in MEC both dual key features of endotoxin tolerance. It dampened the re-stimulation-induced expression of the master regulators of inflammation, *TNF* and *IL1Β*, and increased the basal expression of factors protecting the udder cells against damage (*SAA3*, *TGM3*) and some of those with bactericidal function (*SLPI*, *LAP*, *NOS2A*). Increasing the basal level of protective factors in epithelial cells is a major beneficial aspect of endotoxin tolerance as this will reduce the probability of reinfection by enforcing the bactericidal properties of the alveolar fluid. Enhanced expression of protective factors during ET is well known,^11^ but has not often been considered in the many studies analyzing endotoxin tolerance in professional immune cells. Pam2CSK4 priming enhanced also the re-challenge stimulated induction of the expression the membrane protective factors, to the same or even slightly stronger extent than encountered with LPS. Hence, priming immune functions through the TLR2/6 axis might yield as good an immune protection of the udder against re-colonization as priming with LPS via the TLR4 axis, albeit that the TLR2 signaling cascade is less complex than that of TLR4.^62^

Our data may serve as a platform to validating the physiological relevance of such treatments in a relevant animal model.^5^ Moreover, only such *in vivo* experiments can show if serious problems arise through the reduced capacity of the TLR2 axis mediated priming to confining the potentially harmful induction of the master cytokines (TNF-α, IL-1β) and of NOS2A during re-challenges.

### Blocking histone modifications modified the immune reactivity into an undesired direction

Priming the cells should, upon re-stimulation, dampen the exuberant expression of inflammatory cytokines and, at the same time, result in sustained increased expression bactericidal and membrane protective factors. However, the treatments with inhibitors of histone-modifying enzymes only partly fulfilled these expectations. Our inhibitors of histone modulators substantially quenched the expression of only some of our candidate cytokine- and chemokine-encoding genes (*TNF*, *CCL5*) but not of *IL1Β* or *CXCL8*. This may be owing to the fact that the chromatin at promoters of resting primary immune response genes is known to be in an ‘open’ configuration, with necessary transcription factors having already been recruited so that these genes are poised to react immediately upon an incoming stimulus.^63,64^ Hence, there may be no need for extensive chromatin remodeling to trigger their expression.

However, the general failure of the three classes of inhibitors to increase substantially the basal expression of the protective factors was unexpected and clearly disqualifies them as suitable candidate substances. The demethylase inhibitor S2101 very significantly quenched the basal, as well as the re-challenge-induced expression of all the respective candidate genes. This suggests that their expression may, in part, be confined through repressive histone methylation, such as H3K27me3. Moreover, the particularly strong repressive effect of C646 upon re-stimulating their expression could indicate that *de novo* acetylation of histone H3, for instance at H3K14, might be of key importance for activating their expression. However, we can only speculate about the mechanisms underpinning the effect of these treatments, as our study was not designed to dissect any of the myriad of mechanisms being triggered through differential histone modifications. Such an analysis into the mechanisms would also need to consider potential off-target and side effects of those inhibitors. C646, for example, not only blocks H3 acetylation, but also the function of the p300/CBP transcriptional co-activator. p300 itself is a promiscuous acetyl transferase with more than 75 targets, including NF-κB factors (see Bowers et al.^32^ for a review).

Our study shows that only priming the immune responsiveness of MEC through the TLR axis induced the dual features of endotoxin tolerance, dampening overshooting inflammation and at the same time harnessing the bactericidal and cell-protective functions. ET is an ancient memory of the innate immune system that exists already in teleostean fish.^65^ Obviously, priming immune competence and reactivity through TLR ligands triggers a very complex response network having been evolutionarily streamlined for > 450 million yr, before the radiation of the teleostean fish from all other vertebrates.^66^ It is activated by even older bacterial molecular patterns derived from structural core components of pathogens having functionally been optimized for > 2 billion yr. All TLR signaling converges ultimately in NF- κB activation. A pivotally important TLR-signaling-triggered NF- κB activation for the induction of ET has previously been reported.^23^ However, priming the immune responsiveness of these cells by selectively interfering with a single class of epigenetic regulators will only modulate a sub-section of the mechanisms operating during ET rather than adequately activate the entirety of this regulatory network.

In summary, our study validates that mimetics for bacterial lipopeptides and lipoproteins are capable of inducing the beneficial features of ET in mammary epithelial cells. Their derivatives might therefore serve as promising candidate substances in eliciting a timely limited immune protection in the udder. Our data may serve as a platform for validating the physiological relevance *in vivo*. However, we also show that our selected modulators of epigenetic regulators all failed to harness the cell protective and bactericidal features of ET in MEC.

## Supplementary Material

Supplementary material
