# Supplementary material for: TLR ligands, but not modulators of histone modifiers, can induce the complex immune response pattern of endotoxin tolerance in mammary epithelial cells
Source: Innate Immun. 2016 Dec 5;23(2):155–64. doi: 10.1177/1753425916681076 (PMC5410871; doi:10.1177/1753425916681076)
Supplement: Supplementary material [file INI681076_supplementary_materials.pdf]

Supplementary materials

Toll-like receptor ligands, but not modulators of histone modifiers can induce the complex immune response pattern of endotoxin tolerance in mammary epithelial cells.

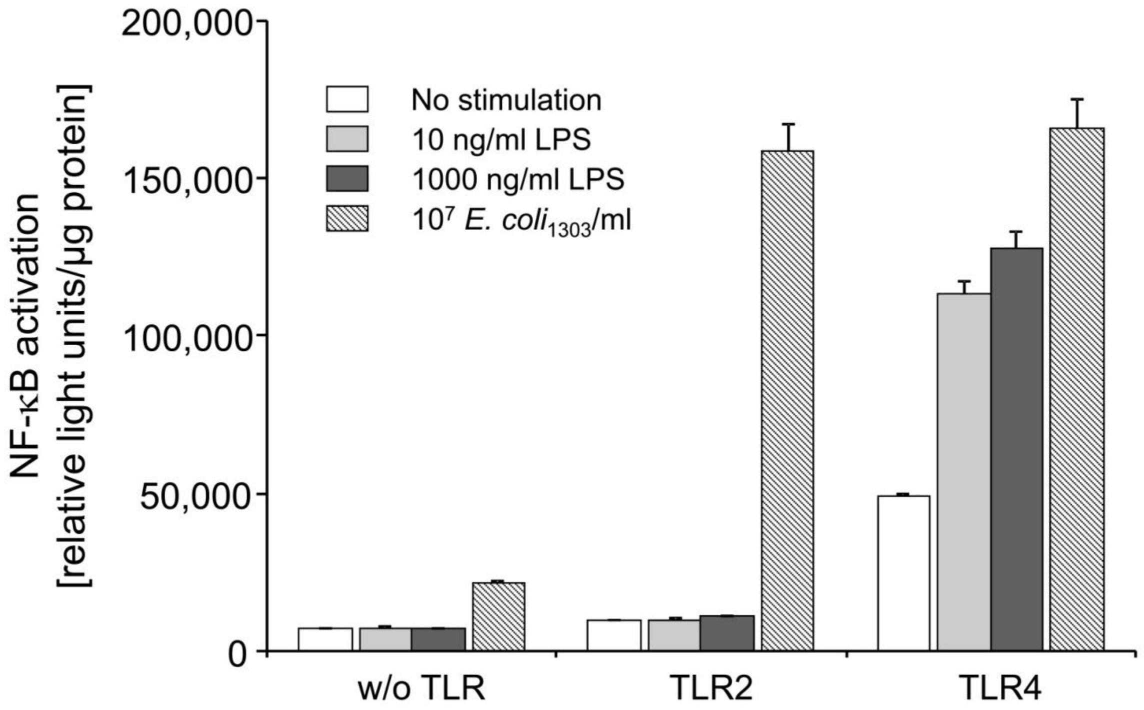

Supplementary figure S1. LPS was free of TLR2 ligands. TLR activation through LPS or heat killed particles of *E. coli* $_{1303}$ was measured in the HEK293 reconstitution system of TLR signaling by the parameter of NF-κB activation (ordinate). Levels of active NF-κB factors were determined with a NF-κB driven ELAM reporter gene construct expressing the Renilla-luciferase. Enzyme activity was expressed as relative light units per µg of protein from the cell lysates. LPS did not activate TLR2 but stimulated TLR4 in a dose dependent fashion. *E. coli* stimulation activated both TLRs.

Methods: The assay system has been described in detail in Yang et al., 2008 Mol Immunol 45(5):1385-97. Briefly: HEK293 cells do not express TLR2 or -4. These cells were transfected (Lipofectamine 2000) with 100 ng of vectors expressing the bovine TLR2 or -4 receptors together with a Renilla-luciferase expressing reporter gene being under the control of the ELAM promoter. This features 5 NF-κB binding sites. The TLR4 assays required co-transfection of CD14 and MD2 expressing constructs (100 ng, each). Twenty-four h after transfection the cell were stimulated for 24 h, as indicated. Subsequently, cells were lysed and the enzyme activity measured (Berthold luminometer). Each transfection was assayed in triplicate.

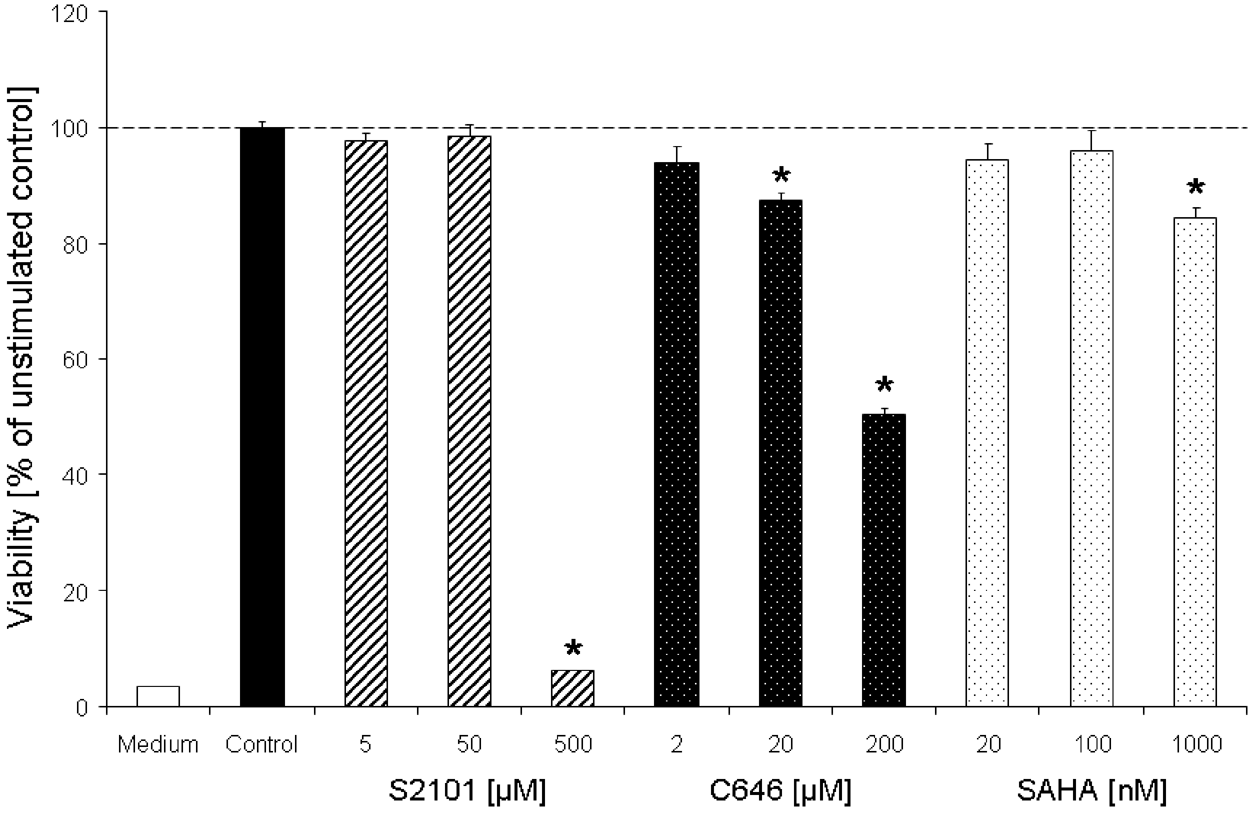

Supplementary figure S2. Influence of selected priming substances on pbMEC viability. Cells were treated for 12 h with different concentrations of the substances as indicated and their viability was determined with the MTT-assay. Columns represent mean values from triplicate assays. Asterisk indicates significant (t-Test, $P < 0.05$) difference to the untreated controls.

Supplementary table S1: List of primers used for RT-qPCR. Sequences of the oligonucleotide primers used for real-time PCR quantification and the pertinent ENSEMBL reference files.

Gene	Ensembl no.	Primer sequence (5´–3´)	Amplificate size (bp)
TNF	ENSBTAG00000025471	CTTCTGCCTGCTGCACTTCG *<br>GAGTTGATGTCGGCTACAACG *	156
IL1A	ENSBTAG00000010349	GGCCAAAGTCCCTGACCTCT<br>CTGCCACCATCACCACATTC	224
IL1B	ENSBTAG00000001321	AACCGAGAAGTGGTGTTCTGC<br>TTGGGGTAGACTTTGGGGTCT	167
IL6	ENSBTAG00000014921	GGAGGAAAAGGACGGATGCT<br>GGTCAGTGTTTGTGGCTGGA	227
CXCL2	ENSBTAG00000027513	GCCAAACCGAAGTCATAGCC<br>TGGAAACCAGCCATTCTCTTC	213
CXCL8	ENSBTAG00000019716	CCTCTTGTTCAATATGACTTCCA<br>GGCCCACTCTCAATAACTCTC	170
CCL2	ENSBTAG00000037811	CGCTGCAACATGAAGGTCTC<br>TGGGGTCTGCACATAACTCCT	244
CCL5	ENSBTAG00000007191	TCCCCATATGCCTCGGAC<br>TCGCACCCACTTCTTCTCTG	229
CCL20	ENSBTAG00000021326	CAGCAAGTCAGAAGCAAGCAA<br>CCCACTTCTTCTTTGGATCTGC	179
NOS2A	ENSBTAG00000006894	ACAGGATGACCCCAAACGTC<br>TCTGGTGAAGCGTGTCTTGG	188
LAP	ENSBTAG00000027225	AGGCTCCATCACCTGCTCCTT<br>CCTGCAGCATTTTACTTGGGCT	182
SLPI	ENSBTAG00000004148	AATGTTGCCTCGACACTTGC<br>TACGCACTGGTCGTCTGTCTC	152
S100A9	ENSBTAG00000006505	CTCAAACAGAAGGCGGGAAA<br>TTGTGTCCAGATCCTCCATGA	225
CD36	ENSBTAG00000017866	CTCCTTCTAAGACTCATCAGATC<br>GAGCCCACAGTTTCGATTGCA	177
CD40	ENSBTAG00000020736	GCAGTGTCTCTTCTGGGGCT<br>GCGGGCACAAATCACAGCA	110
SAA3	ENSBTAG00000022396	CTTTCCACGGGCATCATTTT<br>CTTCGGGCAGCGTCATAGTT	188
TGM3	ENSBTAG00000001785	GCATGGATGTCACCGAAAAG<br>TACACCAGCGACCTTGAACC	179
NFKB1	ENSBTAG00000020270	TCAAACTCCAGAATGGCAGA<br>GAAATCCTCTCTGTTTAGGTTGCTC	179
NFKBIZ	ENSBTAG00000010987	GGCAACAGCAATATGCAGGA<br>AACCTTGGCGGAAATCCAAT	156
SIGIRR	ENSBTAG00000011149	TGAGCAATGGAAGCCTCTATGA<br>AGCTGATGTTCCGGATGGAG	147
JMJD3	ENSBTAG00000048062	CATCCCGTTTGCTTTCCAG<br>ACTGTGCTGCTGACGTTGTG	177
NR4A2	ENSBTAG00000003650	GCCTACAGGTCCAACCCAGT<br>TGCAGGAGAAGGCAGAAATG	163
CLIC1	ENSBTAG00000013533	GTCTCAGTCCGCCTCTTGGT<br>AGAACAACCGCAGGTCGAAT	153

*upper line: forward-, lower line: reverse-primer

Supplementary table S2: Dose-dependent response of gene expression in pbMEC to LPS or Pam2CSK4 pre-stimulation and its effect upon subsequent *E. coli* stimulation. Data survey comprising all genes having been considered.

Gene / function	[ng/ml]	Priming LPS	Priming Pam2CSK4	*E. coli* induction post priming LPS	*E. coli* induction post priming Pam2CSK4
**Cytokines, Chemokines**					
TNF	10	1.6 ± 0.5	**2.6** ± 0.3	**-2.1** ± 0.1	-1.3 ± 0.04
	100	**5.3** ± 0.2	**4.5** ± 0.6	**-2.9** ± 0.2	**-2.2** ± 0.03
	1000	**29.6** ± 4.0	**7.8** ± 0.6	**-5.7** ± 0.1	-1.5 ± 0.1
IL1A	10	**3.0** ± 0.5	1.2 ± 0.02	1.4 ± 0.04	1.3 ± 0.2
	100	**2.9** ± 1.1	0.9 ± 0.1	1.3 ± 0.004	**-2.1** ± 1.0
	1000	**2.9** ± 0.2	1.2 ± 0.2	-1.2 ± 0.2	1.0 ± 0.1
IL1B	10	**3.5** ± 1.2	**4.5** ± 0.7	-1.2 ± 0.1	-1.3 ± 0.1
	100	**4.4** ± 0.8	**4.5** ± 0.1	**-2.0** ± 0.02	-1.7 ± 0.2
	1000	**16.6** ± 0.7	**8.1** ± 1.7	**-4.9** ± 0.2	-1.3 ± 0.1
IL6	10	1.6 ± 0.5	**2.7** ± 0.1	1.2 ± 0.04	1.3 ± 0.02
	100	**2.4** ± 0.4	**2.9** ± 0.4	-1.0 ± 0.1	1.1 ± 0.01
	1000	**3.5** ± 0.1	**4.1** ± 0.4	-1.2 ± 0.1	1.5 ± 0.2
CXCL2	10	1.0 ± 0.2	1.4 ± 0.003	1.1 ± 0.01	-1.2 ± 0.05
	100	1.4 ± 0.2	1.6 ± 0.2	-1.3 ± 0.02	-1.6 ± 0.1
	1000	**2.0** ± 0.03	**2.1** ± 0.6	-1.5 ± 0.2	-1.4 ± 0.2
CXCL8	10	1.8 ± 0.2	**2.3** ± 0.2	1.4 ± 0.01	1.1 ± 0.05
	100	**3.2** ± 0.3	**3.5** ± 0.6	-1.4 ± 0.04	-1.2 ± 0.1
	1000	**4.1** ± 0.2	**5.8** ± 0.2	-1.7 ± 0.3	-1.2 ± 0.5
CCL2	10	0.8 ± 0.02	0.9 ± 0.04	-1.8 ± 0.04	-1.4 ± 0.1
	100	1.0 ± 0.1	1.0 ± 0.1	**-2.1** ± 0.1	-1.9 ± 0.1
	1000	1.3 ± 0.1	1.5 ± 0.1	**-2.0** ± 0.1	-1.7 ± 0.4
CCL5	10	**21.5** ± 8.0	**6.6** ± 0.5	-0.5 ± 0.05	-1.0 ± 0.4
	100	**33.8** ± 14.4	**3.1** ± 0.3	**-2.9** ± 0.6	**-3.4** ± 0.5
	1000	**8.0** ± 0.5	**3.9** ± 3.5	**-4.7** ± 1.6	**2.0** ± 0.05
CCL20	10	**3.0** ± 1.3	**3.5** ± 0.7	-1.7 ± 0.02	-1.5 ± 0.03
	100	**4.0** ± 0.8	**5.0** ± 0.9	**-2.3** ± 0.1	-1.9 ± 0.2
	1000	**8.1** ± 0.2	**6.7** ± 0.4	**-5.8** ± 0.6	-1.5 ± 0.2
**Bactericidal effectors**					
NOS2A	10	**2.2** ± 1.2	**4.6** ± 1.8	**-2.4** ± 0.3	-1.2 ± 0.2
	100	**2.1** ± 0.4	**2.3** ± 0.2	**-4.8** ± 0.4	-1.5 ± 0.2
	1000	**2.6** ± 0.1	**4.3** ± 1.2	**-12.8** ± 1.9	-1.3 ± 0.1
LAP	10	**5.3** ± 0.5	**5.8** ± 3.6	**2.0** ± 0.1	**2.2** ± 0.5
	100	**6.3** ± 1.4	**2.0** ± 0.3	1.4 ± 0.2	**2.2** ± 0.6
	1000	**4.0** ± 1.4	**3.6** ± 0.1	1.6 ± 0.3	1.7 ± 0.2

SLPI	10	**2.6** ± 0.6	**2.9** ± 0.3	**3.5** ± 0.4	**4.9** ± 0.5
	100	**3.1** ± 0.7	**3.2** ± 0.2	1.7 ± 0.1	**5.5** ± 1.5
	1000	**3.1** ± 1.1	**5.5** ± 0.6	**2.3** ± 0.7	**3.9** ± 0.4
S100A9	10	**3.1** ± 0.5	1.2 ± 0.02	1.4 ± 0.04	1.3 ± 0.2
	100	**2.9** ± 1.1	0.9 ± 0.1	1.3 ± 0.004	**-2.1** ± 1.0
	1000	**2.9** ± 0.2	1.2 ± 0.2	-1.2 ± 0.2	1.0 ± 0.1
**By-stander factors for pathogen induced signaling**					
CD36	10	1.2 ± 0.2	1.1 ± 0.03	1.4 ± 0.1	1.4 ± 0.1
	100	1.2 ± 0.2	0.9 ± 0.1	0.9 ± 0.001	1.0 ± 0.04
	1000	0.8 ± 0.003	1.2 ± 0.3	1.0 ± 0.03	**2.0** ± 0.4
CD40	10	1.1 ± 0.1	1.2 ± 0.1	1.0 ± 0.01	1.1 ± 0.01
	100	1.4 ± 0.2	1.1 ± 0.1	0.8 ± 0.1	1.1 ± 0.1
	1000	1.2 ± 0.1	1.3 ± 0.03	0.9 ± 0.1	1.2 ± 0.2
**Acute phase proteins, membrane protection**					
SAA3	10	**20.7** ± 0.6	**11.3** ± 0.6	**2.1** ± 0.1	**2.2** ± 0.2
	100	**21.4** ± 2.9	**9.6** ± 0.9	1.4 ± 0.1	1.6 ± 0.1
	1000	**14.6** ± 1.3	**11.0** ± 0.5	1.0 ± 0.2	1.7 ± 0.2
TGM3	10	1.9 ± 0.2	**2.6** ± 0.1	**2.6** ± 0.2	**2.9** ± 0.01
	100	**2.1** ± 0.3	**3.0** ± 0.2	1.8 ± 0.0	**3.6** ± 0.1
	1000	**2.0** ± 0.2	**4.3** ± 0.3	**2.5** ± 0.3	**5.6** ± 0.2
**Relevant transcription factors and regulators**					
NFKB1	10	1.4 ± 0.3	1.8 ± 0.03	1.4 ± 0.01	1.3 ± 0.1
	100	1.5 ± 0.3	1.0 ± 0.1	0.9 ± 0.1	1.0 ± 0.2
	1000	1.1 ± 0.1	1.4 ± 0.3	1.2 ± 0.3	1.5 ± 0.1
NFKBIZ	10	1.4 ± 0.2	1.5 ± 0.1	1.2 ± 0.03	-1.3 ± 0.1
	100	1.4 ± 0.2	1.3 ± 0.1	0.8 ± 0.1	-1.5 ± 0.1
	1000	1.6 ± 0.2	1.9 ± 0.2	0.8 ± 0.2	-1.3 ± 0.1
SIGIRR	10	1.4 ± 0.1	1.2 ± 0.04	1.1 ± 0.1	1.4 ± 0.1
	100	1.3 ± 0.2	1.0 ± 0.1	0.9 ± 0.0	1.1 ± 0.01
	1000	0.9 ± 0.1	1.0 ± 0.1	1.0 ± 0.2	1.2 ± 0.2
**Factors involved in epigenetic mechanisms**					
JMJD3	10	1.0 ± 0.1	1.1 ± 0.001	1.0 ± 0.1	0.9 ± 0.1
	100	1.0 ± 0.2	0.9 ± 0.04	0.8 ± 0.0	1.0 ± 0.03
	1000	1.0 ± 0.1	0.9 ± 0.04	1.0 ± 0.0	1.0 ± 0.03
NR4A2	10	1.5 ± 0.3	1.8 ± 0.05	1.2 ± 0.0	1.4 ± 0.1
	100	1.7 ± 0.3	1.3 ± 0.1	0.9 ± 0.0	1.3 ± 0.1
	1000	1.4 ± 0.1	1.5 ± 0.3	1.0 ± 0.2	1.5 ± 0.02

Values are fold changes of mRNA concentrations relative to un-stimulated (priming) or *E. coli* challenged cells (*E. coli* induction post priming). Bold numbers indicate changes larger than 2-fold. Mean values ± SEM from duplicate determinations.
